# Evaluation of long-term immunity and protection against *T. gondii* after immunization with multivalent recombinant chimeric *T. gondii* proteins

**DOI:** 10.1038/s41598-023-40147-z

**Published:** 2023-08-10

**Authors:** Maciej Chyb, Bożena Dziadek, Katarzyna Dzitko, Bartłomiej Tomasz Ferra, Malwina Kawka, Lucyna Holec-Gąsior, Justyna Gatkowska

**Affiliations:** 1 Department of Molecular Microbiology, Faculty of Biology and Environmental Protection, University of Lodz, Banacha 12/16, 90-237 Łódź, Poland; 2 Bio-Med-Chem Doctoral School of the University of Lodz and Lodz Institutes of the Polish Academy of Sciences, Faculty of Biology and Environmental Protection, University of Lodz, Banacha 12/16, 90-237 Łódź, Poland; 3 Department of Tropical Parasitology, Institute of Maritime and Tropical Medicine in Gdynia, Medical University of Gdańsk, Powstania Styczniowego 9B, 81-519 Gdynia, Poland; 4 Department of Molecular Biotechnology and Microbiology, Faculty of Chemistry, Gdańsk University of Technology, Narutowicza 11/12, 80-233 Gdańsk, Poland

**Keywords:** Vaccines, Protein vaccines, Parasitology

## Abstract

Toxoplasmosis caused by the opportunistic, cosmopolitan protozoan *Toxoplasma gondii* is one of the most common parasitoses in the world. Although it may prove dangerous or even fatal for immunocompromised individuals, immunoprophylaxis for humans is still nonexistent. Thus, the aim of the current work was to assess the ability of two immunogenic recombinant chimeric *T. gondii* proteins, SAG2-GRA1-ROP1 (SGR) and SAG1-MIC1-MAG1-GRA2 (SMMG), selected in previous experiments to induce long-lasting immunity when administered with a safe adjuvant. Thus, the determination of immunological parameters and parasite challenge were performed both two weeks after the last boost injection and 6 months postvaccination. Both experimental vaccines triggered specific humoral and cellular responses in immunized C3H/HeOuJ male mice, characterized by the production of specific IgG (IgG1/IgG2a) antibodies in vivo and the synthesis of key Th1/Th2 cytokines by *Toxoplasma* lysate antigen-stimulated splenocytes in vitro. Although the levels of specific antibodies and cytokine release were in most cases lower six months postimmunization, the protection rates conferred by the vaccination were comparable regardless of the time after the administration of the last vaccine dose. The results indicate that both preparations induce long-lasting immunity, which makes them attractive candidates for further research aimed at boosting their immunogenicity and immunoprotective capacity.

## Introduction

*Toxoplasma gondii* is an intracellular protozoan parasite and an etiological factor of toxoplasmosis in humans and animals, which is one of the most common parasite infection in the world. This parasite is known for its complex life cycle and the only definitive hosts of this protozoan are members of the *Felidae* family, in which *T. gondii* undergoes sexual reproduction and asexual multiplication in all warm-blooded animals. This parasite has multiple routes of transmission, such as raw or undercooked meat, soil, water, vegetables, and fruits contaminated with oocysts from cat feces, congenital or organ transplants. After oral infection, *T. gondii* invades enterocytes in the small intestine and multiplies asexually. Tachyzoites cause cell rupture and invade other cells and are responsible for the acute phase of infection. They can transform into bradyzoites and create tissue cysts under pressure from the host immune system^1^. The seroprevalence of infection varies depending on geographical regions. In Europe, Asia, and North America, it is reported in approximately 10–30% of the population, while in South America and Africa, it is reported in up to 95%^2^. Infection with this parasite is largely asymptomatic or with flu-like symptoms. However, for immunodeficient people (AIDS patients, people on immunosuppressive therapy), it poses a serious health risk, causing encephalitis or pneumonitis. In women with primary infection, tachyzoites can cross the placenta into the fetus, and due to the underdeveloped immune system, they propagate asexually without disturbance which may lead to malformations (such as hydrocephalus) of the fetus or even miscarriage. This can also be a huge problem in the case of goat and sheep breeding since it can lead to economic losses^3^. Because of its affinity to nerve cells, *T. gondii* is linked to various neurological disorders, such as schizophrenia and even glioma^4,5^. It is also known that *T. gondii* causes behavioral changes in animals. Infection with *T. gondii* is usually treated by a combination of pyrimethamine, sulfadiazine or trimethoprim^6^. Unfortunately, this therapy has limitations as it does not act against bradyzoites in tissue cysts and therefore, there is a risk of reactivation of infection. Additionally, these chemotherapeutics cause a number of side effects and moreover, increasing resistance against these drugs is observed^7^. One solution to overcome this problem is the search for new chemical compounds with potential anti-*T. gondii* activity, such as thiosemicarbazide, thiazolidinone or thiadiazole derivatives, which show a high potential to inhibit the development of this parasite in vitro^8–10^. Another option is protection induced by vaccination, which would prevent the infection and its consequences. Over the past years, researchers have focused their attention on potential candidates for *T. gondii* vaccines. The most studied technologies were inactivated vaccines, live attenuated vaccines, protein vaccines and DNA vaccines. To date, there have been many attempts to develop an effective vaccine against *T. gondii*, however due to complex life cycle of this parasite, no effective vaccine has been developed, although some strategies are more promising than others^11–13^. The only commercially available vaccine is Toxovax, which contains live attenuated tachyzoites of the S48 strain^3,14^. This vaccine is licensed in Europe and is used to prevent miscarriage in sheep but is not able to prevent parasite infection completely in the case of cyst-forming strains^15^. Unfortunately, this vaccine has several disadvantages, e.g., storage conditions, expensive production, adverse effects and risk of reversion to pathogenic form. This is why more attention is directed to new vaccine technologies using single or multiantigen approaches. Due to intense studies, more than 1360 protein families of *T. gondii* have been described^2^. Multiple studies have focused on antigens crucial in the life cycle of *T. gondii* that are involved in parasite adhesion, invasion and replication in target cells. Such antigens include rhoptry antigens (ROP), dense granule antigens (GRA), surface antigens (SAG) and microneme antigens (MIC). The main role of SAG surface antigens of *T. gondii* is mediating attachment to host cells and interfering with the host immune system. MIC antigens play a role in parasite adhesion and penetration of host cells by forming microneme protein complexes. ROP antigens are secreted from the bulbs of rhoptry organelles, and their main function is the formation of parasitophorous vacuoles. GRA antigens are secreted from high-density granules in the early stage of toxoplasmosis and they take part in the structural modification of parasitophorous vacuoles^16^. Not all antigens are expressed in every stage of the *T. gondii* life cycle. The expression of antigens varies even in a particular antigen group, e.g., in the MIC protein family, MIC1 antigen is expressed in tachyzoites and bradyzoites, while MIC3 is expressed in all three infectious stages^17^. There are also those antigens that have been thought to be stage specific, e.g., matrix antigen 1 (MAG1), which is usually associated with the bradyzoite stage but is also present in tachyzoites^18^. Therefore, the selection of antigens seems to be the key to developing an effective vaccine; moreover, using multiple antigens as vaccines seems to be more effective than the use of only one^14,19^.

The aim of this study was to evaluate the long-term persistence of protective immunity following immunization with two experimental vaccines based on recombinant tri- SAG2-GRA1-ROP1 (SGR) and tetravalent SAG1-MIC1-MAG1-GRA2 (SMMG) chimeric *T. gondii* recombinant proteins in a murine model with a preclinical grade AddaVax squalene-based oil-in-water nano emulsion adjuvant. The chimeric proteins selected for this study were previously tested and showed a high immunoprophylactic potential when administered with incomplete Fraud's adjuvant. In this work, we used the AddaVax adjuvant, which is the equivalent of MF59 used in commercially available flu vaccines for humans^20^.

## Results

### Activation of NF-κB pathway in human monocytes

**Figure 1:**
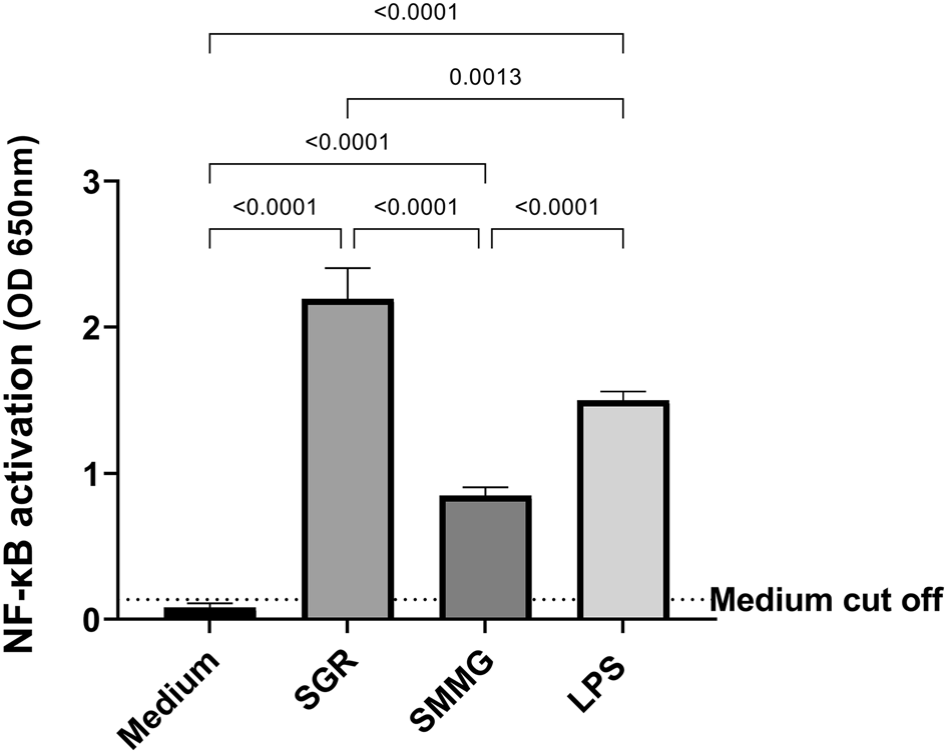
NF-κB induction in THP1-Blue human monocytes exposed to SGR or SMMG recombinant antigens. Figure shows data of representative experiment (from two independent experiments). Values were presented as mean of six repeats and standard deviation. Cut off value was calculated of mean value of cells cultured in medium alone + 2*standard deviation. Statistical comparison was performed using Welch and Brown-Forsythe one way ANOVA followed by Dunnet`s post hoc.

To assess the recombinant antigens potential to elicit a proinflammatory response of monocytes and macrophages, the modified THP1-Blue human monocytes with NF-κB-inducible SEAP reporter construct were used. Responses in the NF-κB pathway activation, of human THP1 monocytes, to recombinant chimeric *T. gondii* antigens stimulation, were assed (Fig. 1). The tested recombinant antigens proved to have no toxic effect on stimulated cells as proved by the resazurin reduction assay (Supplementary Fig. 1). Statistical analysis showed that both antigens cause significant reaction from human monocytes, leading to high levels of proinflammatory NF-κB pathway activation when compared to unstimulated cells (medium). SGR antigen turned out to be a much better activator of NF-κB pathway than SMMG, causing statistically significant activation compared to both SMMG antigen and positive control LPS.

### Immunoglobulin production

**Figure 2:**
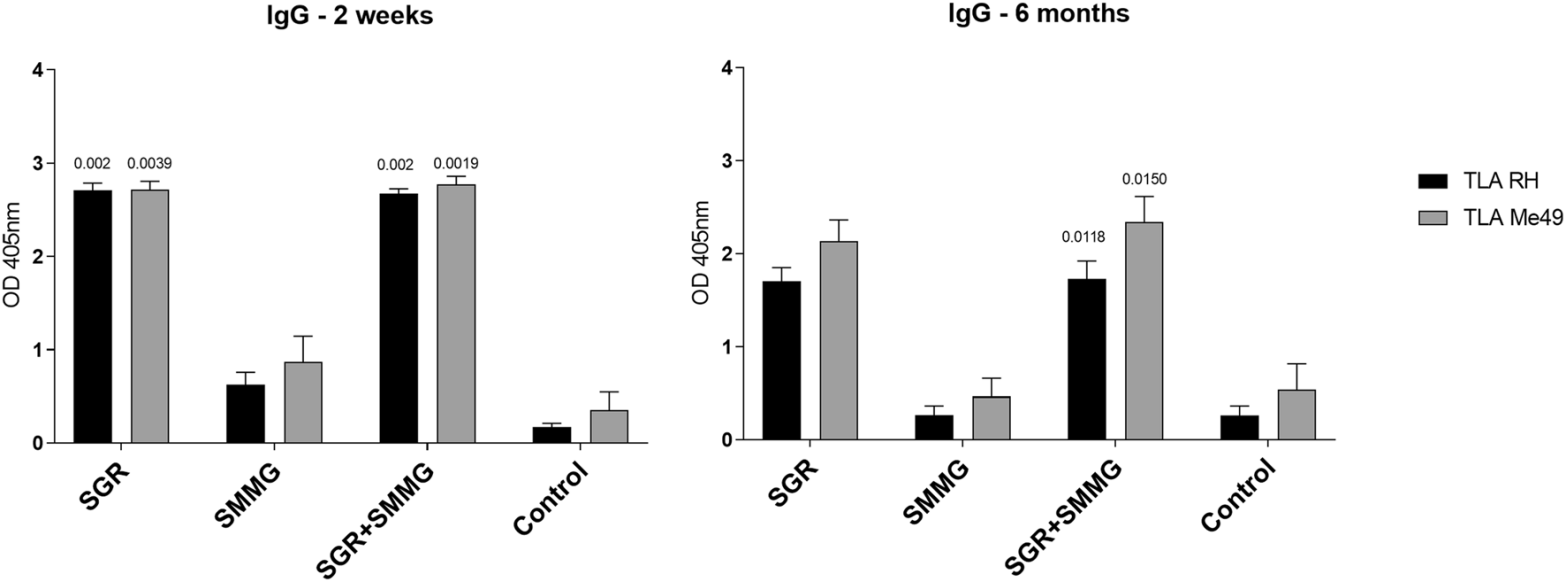
Reactivity of IgG antibodies specific to TLA of RH and Me49 *T. gondii* strains present in the sera of immunized (SGR, SMMG, SGR + SMMG) and control (PBS) mice. Serum dilution 1:100. Values are presented as the mean and standard deviation. Statistics were calculated using Kruskal-Walli’s test followed by Two-stage linear step-up procedure of Benjamini, Krieger and Yekutieli post hoc, numbers above bars represents q value in regard to appropriate control group.

Vaccination of C3H/HeOuJ mice with the recombinant chimeric *T. gondii* proteins SGR and SMMG triggered a humoral immune response, resulting in statistically significant production of TLA-specific IgG class antibodies compared to the control group (Fig. 2), regardless of the time post infection or TLA used in the test. The highest level of specific antibodies was observed in both the SGR and SGR + SMMG vaccinated groups, both 2 weeks and 6 months after the last immunization. In the SGR group, 6 months after the last dose of vaccine, no significant difference in IgG levels compared to the control group could be demonstrated because two of four mice used for serology died during the 6-months period. A non-significant decrease in IgG antibody levels was observed over time when comparing the 2-week and 6-months experiments: TLA RH q value = 0.2465, and TLA Me49 q value = 0.3134, for both the SMMG and SGR + SMMG group.

**Figure 3:**
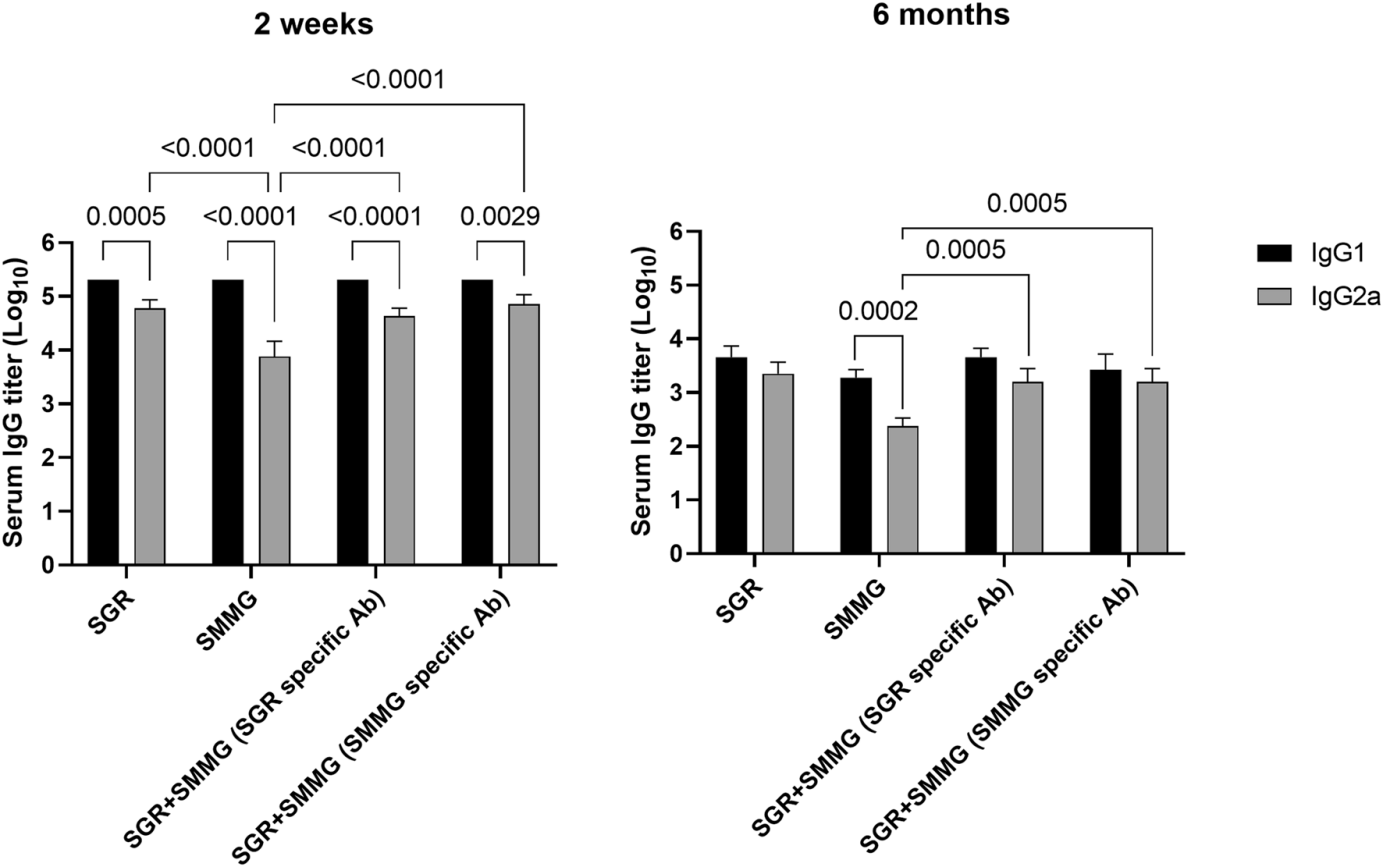
Log10 transformed IgG1 and IgG2a antibody titers. Values are presented as the mean and standard deviation. Statistical comparisons were performed using two-way ANOVA, followed by Tukey's multiple comparisons test.

To assess the type of the immune response, titers of IgG1 (Th2) and IgG2a (Th1) subclass antibodies specific to antigens used for vaccination present in the sera of immunized mice were determined. The test included a negative control, the sera of unvaccinated mice without antigen-specific antibodies that did not reach the OD threshold of 0.3 at a 1:100 dilution. Titration graphs can be viewed in supplementary materials (Supplementary Fig. 2). As shown in Fig. 3 and Table 1, the IgG1 subclass was dominant in all experimental groups. In the case of the IgG1/IgG2a titer ratio, we did not notice significant differences between groups, except for the SMMG group in the 2-week experiment, where we observed a low IgG2a antibody titer, which resulted in a high IgG1/IgG2a ratio, even reaching 64:1 (Table 1). Importantly, we noticed an intense decrease in the IgG1 subclass antibody titer over time, comparing the 2-week to 6-months experiments, in every group of vaccinated mice, while the IgG2a antibody subclass titer decreased to a lesser extent.

**Table 1:** Titers of IgG1 and IgG2a antibodies in the sera of vaccinated mice.

Mouse/group	IgG1/IgG2a titer 2 weeks after last dose			IgG1/IgG2a titer 6 months after last dose		
	IgG1	IgG2a	IgG1/IgG2a	IgG1	IgG2a	IgG1/IgG2a
SGR						
Mouse 1	> 204,800	51,200	> 4/1	6400	1600	4/1
Mouse 2	> 204,800	102,400	> 2/1	3200	3200	1/1
Mouse 3	> 204,800	51,200	> 4/1			
Mouse 4	> 204,800	51,200	> 4/1			
SMMG						
Mouse 1	> 204,800	12,800	> 16/1	1600	400	4/1
Mouse 2	> 204,800	6400	> 32/1	1600	200	8/1
Mouse 3	> 204,800	12,800	> 16/1	1600	200	8/1
Mouse 4	> 204,800	3200	> 64/1	3200	200	16/1
SGR + SMMG (SGR specific Ab)						
Mouse 1	> 204,800	51,200	> 4/1	3200	1600	2/1
Mouse 2	> 204,800	51,200	> 4/1	6400	800	8/1
Mouse 3	> 204,800	25,600	> 8/1	3200	3200	1/1
Mouse 4	> 204,800	51,200	> 4/1	6400	1600	4/1
SGR + SMMG (SMMG specific Ab)						
Mouse 1	> 204,800	51,200	> 4/1	1600	800	2/1
Mouse 2	> 204,800	102,400	> 2/1	3200	1600	2/1
Mouse 3	> 204,800	51,200	> 4/1	1600	3200	1/2
Mouse 4	> 204,800	102,400	> 2/1	6400	1600	4/1

**Figure 4:**
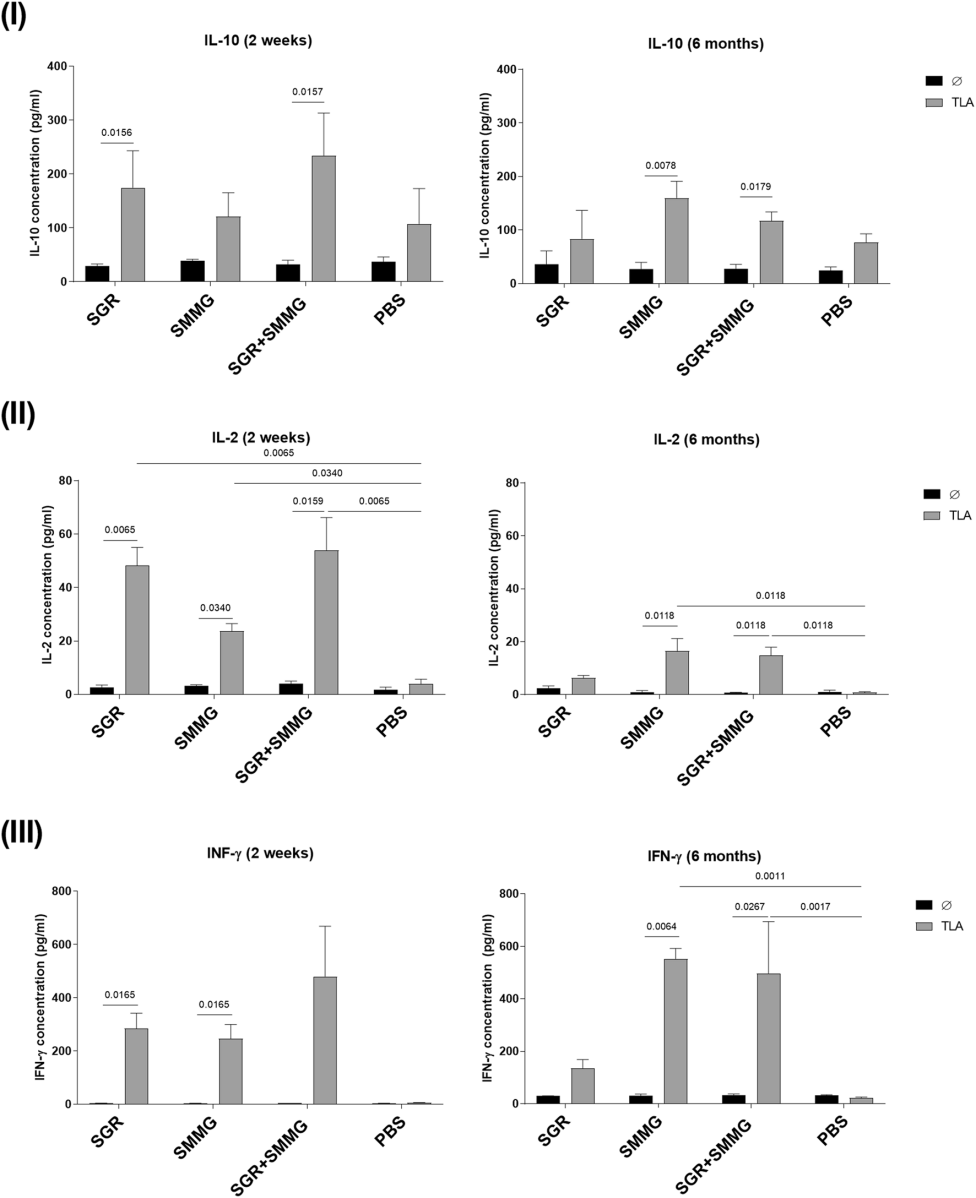
In vitro levels of IL-10 (**I**), IL-2 (**II**), and IFN-γ (**III**) secreted by splenocytes in the control, SGR, SMMG and SGR + SMMG-vaccinated groups stimulated with native TLA antigen. Ø-unstimulated cells, TLA-cells stimulated with TLA of the RH strain. Values are presented as the mean and standard deviation (n = 4, except for the SGR group in 6 months scenario, n = 2). Comparison of TLA treated and untreated cells, or TLA treated study group cells and PBS group cells was performed using the Kruskal-Walli’s test followed by Two-stage linear step-up procedure of Benjamini, Krieger and Yekutieli post hoc.

Statistical analysis showed that 2 weeks after the last immunization, for all study groups, the titer of the IgG1 antibody subclass was statistically higher than that of the IgG2a antibody subclass (Fig. 4). In the case of 6 months after the last vaccine dose, this can only be observed in the case of the SMMG group. Furthermore, it was shown that in both experiments (2 weeks and 6 months), the IgG2a antibody titer of the SMMG group was significantly lower than that of every other vaccination group. It should be mentioned that there were no differences in IgG1 and IgG2a antibody titers in the SGR + SMMG group if we assessed antibody reactivity to SGR or SMMG antigen.

### Cytokine assay

As part of assessing the immune response polarization toward Th1 and Th2 lymphocyte cell types, we defined the profile of secreted cytokines important in cellular and humoral immunity in splenocytes of vaccinated and control mice stimulated with the native *T. gondii* antigen (Fig. 4).

In all study groups, stimulation of vaccinated mouse splenocytes with TLA caused increased secretion of cytokines associated with the Th1-type immune response, such as IFN-γ and IL-2. It is most noticeable that 2 weeks after the last vaccine dose, significantly higher cytokine concentrations were observed for immunized groups compared to unstimulated cells (IL-2 for both experiments, INF-γ for both experiments) and cells stimulated with TLA derived from control mice (IL-2 for both experiments, INF-γ for 6 months experiment). This was not observed for the SGR group in the 6-months experiment. IL-10 concentrations were not significantly higher than in control group in any scenario, there were also no differences in IL-10 concentrations between 2 weeks and 6 months experiments. Generally, there was a decrease in IL-2 cytokine concentration when comparing the 2-week and 6-months experiments in every experimental group, especially in case of SGR + SMMG group (q value = 0.0110). In the case of IFN-γ, this was only observed for the SGR group, on the other hand concentration of this cytokine increased significantly in case of SMMG group (q value = 0.0233).

### Decrease in cyst burden

**Figure 5:**
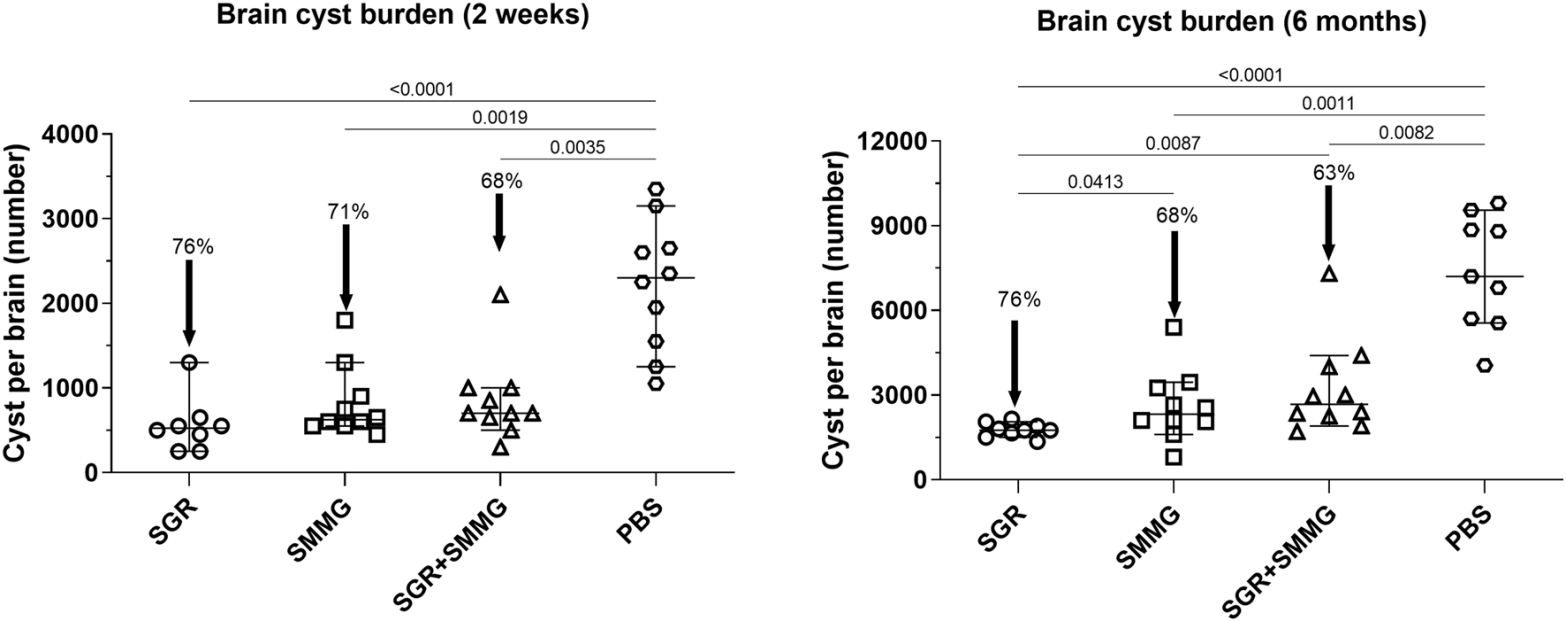
Cyst number per brain in the immunized and control groups. Values are presented on a dot plot with the median and 95% CI. Arrows indicate percent decrease in group cyst median in regard to control. Statistical comparison between study groups or PBS group was calculated using Kruskal-Walli’s test followed by Two-stage linear step-up procedure of Benjamini, Krieger and Yekutieli post hoc.

Vaccination of mice with both experimental chimeric *T. gondii* proteins resulted in the persistence of a high level of immune protection, defined by a decrease in brain cyst burden, in the case of *T. gondii* challenge both 2 weeks and 6 months after the last vaccine dose (Fig. 5). In both experiments, mice were infected for one month prior to sampling. Generally, protection against chronic toxoplasmosis, achieved by vaccination, was approximately 70%. The best result was obtained with SGR protein, with the highest 76% decrease in brain cyst burden, in both the 2-week and 6-months experiments. Differences between study groups were especially marked in 6 months experiment, where cyst burden of SGR group was significantly lower than both SGR + SMMG q value = 0.0087 and SMMG q value = 0.0413 groups. In the case of the SMMG group, we obtained 71% and 68% decrease in cyst burden, while SGR + SMMG caused a decrease in cyst burden by 68% and 63%, respectively, for the 2-week and 6-months experiments.

## Discussion

As mentioned earlier, no effective, commercially available vaccine has yet been developed to prevent horizontal transmission of *T. gondii*, despite many research attempts and methods used. There are several reasons why developing an effective anti-toxoplasmosis vaccine is such a challenge. One of the problems in the development of a vaccine against *T. gondii* is the lack of a unified vaccination schedule, for example, in terms of the strain used or the infectious dose. A high impact of strain variation on immunogenicity is observed. Additionally, some strains, such as RH, propagating in an in vitro laboratory environment no longer match the native strains^21^.

The second most important reason is the multistage life cycle of the parasite, in which we observe a panel of different protein expression levels during particular stages. This poses a threat that the immune response against a specific stage of the life cycle may not be effective against infections caused by other stages. This is why using a reverse vaccinology approach for vaccine development and intense studies on *T. gondii* proteomics are essential. A robust strategy seems to be using a cocktail of multistage antigen vaccines or designing fusion proteins that consist of them. Another inconvenience related to the life cycle of *T. gondii* is a latent stage. Tachyzoites under pressure from the immune system undergo a change to slowly dividing bradyzoites enclosed within a tissue cyst. In this way, the parasites are protected from the immune system and can wait for more favorable conditions and proceed with reinfection^17^.

Another problem is the ability of this parasite to evade and manipulate the host immune system signaling pathways. For example, one of the dense granule proteins, TgIST, can block STAT1-dependent proinflammatory gene expression and thus IFN-γ production. ROP16 secreted from rhoptry organelles into the host cell can promote the Th2-type response by sustained activation of STAT3 and STAT6, resulting in a decrease in IL-12 production^21,22^. Neither of these effector protein functions is beneficial for pathogen clearance. The effectiveness of the immune response to *T. gondii* infection depends on how strong a specific Th1 response with antigen-specific CD8+ T cells is induced. These activated cells produce IFN-γ, which also plays a key role in killing *T. gondii* and/or suppressing its development^23^.

It should be underlined how important it is to consider how long the immune protection will last after vaccination. In most in vivo vaccine studies, *T. gondii* challenge is performed 2 weeks after the last vaccine dose. There are some examples in which challenge is performed 8 weeks after the last immunization with chimeric antigen, but not longer than that^24^. Therefore, potential vaccines should be tested to determine how long the generated immunity will last since the induction of immune memory is an important feature of vaccine-induced protection^25^.

To date, dozens of antigens and antigen compositions have been tested for their vaccine potential in the prophylaxis of toxoplasmosis, with different effectiveness^26–28^. Because none of the single antigen vaccines could induce satisfactory immune protection, more researchers have focused on multiantigenic vaccines containing a mix of promising protein antigens or designed chimeric proteins. This approach can overcome the generally weak immunogenicity of small proteins and induce different life cycle stage-specific protection^2^.

In this work, we assessed the immunoprotective and immunogenic potential of tri- SAG2-GRA1-ROP1 and tetravalent SAG1-MIC1-MAG1-GRA2 chimeric *T. gondii* proteins in a murine model with a parasite challenge performed 2 weeks and 6 months after the last vaccine dose, which allowed us to evaluate the persistence of protective immunity induced by the experimental vaccines. Proteins were emulsified with a squalene-based oil-in-water nanoemulsion AddaVax, similar to MF59, which has been licensed in Europe for flu vaccines and is an equivalent more suitable for use in vaccines than the purely experimental incomplete Freund’s adjuvant (IFA). Both chosen for the study antigens were capable of activating the NF-κB pathway in human THP1 monocytes, with SGR triggering higher response compared to SMMG chimeric protein and were further evaluated for the persistence of the immune response induced by their administration.

Due to the intracellular life cycle of *T. gondii*, major factors in the immune response against infection are cytotoxic lymphocytes Tc and helper Th1, which are involved in the cellular response and secrete cytokines, especially IFN-γ. The production of specific antibodies also plays a role because anti-*T. gondii* antibodies can block the ability of the parasite to invade cells and cause immune-induced cytotoxic effects^23^.

This is why immune parameters were assessed to evaluate the immunogenicity of the tested vaccines. The SGR vaccine induced high production of specific to native *T. gondii* antigen antibodies, which lasted even 6 months after the last dose of vaccine. The antibody level obtained in the SMMG group was noticeably lower than that in the SGR group, which is why in the SGR + SMMG group, the high level of IgG antibodies may be due to the SGR addition to the vaccine composition. However, if we look at IgG1 and IgG2a titers, we can see that the results do not differ regardless of whether antibodies specific to either SGR or SMMG are assessed. This might suggest a synergistic immune response that resulted in a similar level of IgG1 and IgG2a antibodies specific for both vaccine antigens. Moreover, the SMMG-specific IgG2a antibody titer was significantly higher in the SGR + SMMG group than in the SMMG group. The high level of protection in the SMMG group, in contrast to the obtained IgG antibody level, only underlines the important role a cellular response plays in fighting this parasite infection.

One of the most important observations was that although both IgG1 and IgG2a titers dropped after 6 months, it was especially noticeable in the case of IgG1 antibodies, which are a Th2 response marker, while the IgG2a titer, a Th1 marker, decreased to a lesser extent. This phenomenon may partly explain why, even 6 months post-vaccination, the same decrease in cyst burden was obtained.

Selected cytokines, typically evaluated as indicators of Th1 (IFN-γ and IL-2) and Th2 (IL-10) response, produced by the splenocytes of vaccinated and control animals were also assessed to confirm the polarization of the obtained immune response after vaccination^29^. Interleukin 2, with its pleiotropic effect, acts on the proliferation of Tc CD8 + cells, NK cells, B cells and Treg cells. It also plays an important role in the activation and cytotoxicity of Tc CD8 + and NK cells. This cytokine is mostly produced by antigen recognizing Th1 lymphocytes but also to a lesser extent by Tc cells^30^. In our 2-week experiment, TLA induced a high level of IL-2 production by the splenocytes of vaccinated mice compared to PBS. This finding demonstrates a strong response from Th1 helper cells. The highest concentration was observed in the SGR and SGR + SMMG groups. In the case of the 6-months experiment, we detected IL-2 in higher amounts than in the control in all vaccinated mice, and differences proved significant only in the case of the SMMG and SGR + SMMG groups, besides SGR group due to the number of animals. As mentioned in the results section, there was a decrease in IL-2 levels when comparing the 2-week to 6-months experiments. This might suggest that the population of Th1 cells is lower after 6 months of vaccination. At the same time, we observed the release of IFN-γ after both 2 weeks and 6 months in all groups, which could be because of the high population of activated T and NK cells. As mentioned above, IFN-γ is important in the successful eradication of this parasite infection, so a high level is mostly desirable.

While IL-10 can be beneficial to prevent the development of immunopathology in toxoplasmosis^31^, in most cases, it inhibits the immune response by downregulating IL-2 and IFN-γ production by Th1 cells and inhibiting native Th-cell differentiation into Th1 cells. This cytokine is produced by Th2 and Treg cells. In our experiments, we did not observe statistically significant production of this cytokine compared to the PBS group, which should be a good indication of the lack of inhibition of the Th1 response.

Finally, vaccination of mice resulted in a high decrease in brain cyst number after *T. gondii* challenge, ranging from 60 to 76% even 6 months post-challenge. The best results were obtained using SGR antigen (76%), which corresponds to the results of our previous experiments, where SGR antigen administered with Freund’s incomplete adjuvant also proved the most efficient in preventing tissue cyst formation^32^. Although the IFA is a potent adjuvant it can only be used as experimental tool, thus in this work aimed at the assessment of the long-term immunity we used an equivalent of commercially used adjuvant. It is especially important since tested preparations, if fully efficient, are meant to be administered to animals/humans and that can only be done with safe approved adjuvants. The percentage of cyst number decrease compared to the PBS group reflects the level of obtained protection. Unfortunately, even the administration of both recombinant chimeric proteins concomitantly did not result in higher decrease in cyst burden and thus, the results were similar for the SMMG and SGR + SMMG groups, which suggests that the addition of SMMG to the mix did not increase the effectiveness of vaccination. This phenomenon has been reported previously with plasmid immunization^33^. The most important observation is that the results of decrease in cyst burden did not differ between the two experiments, where animals were infected either 2 weeks or 6 months after the last vaccine dose. This suggests that the immunity induced by the experimental vaccine persisted for 6 months, which is a long period considering mouse lifespan of about 2 years.

Interestingly, results of antibody evaluation, most importantly IgG2a level, cytokine production in 2 weeks experiment and decrease in cyst burden seem to correspond to SGR antigen greater, compared to SMMG, capacity to activate NF-κB in monocyte cell line in vitro. Indeed one of the most important roles of nuclear factor kappa B is in the immune system, since numerous cellular processes, e.g. initiation of adaptive immune response, are regulated by NF-jB-responsive genes^34,35^. Although innate responses can be quite effective at the beginning of infection only the activation of adaptive response can trigger robust and durable immune response. NF-κB is required for the production of IFN-γ and inducible nitric oxide synthase (iNOS), which are necessary for resistance to intracellular pathogens, e.g., *T. gondii*^34,36^. Furthermore, NF-κB_2_ knockout mice due to increased lymphocyte apoptosis cannot maintain the T cell response needed for long-term resistance to *T. gondii* infection^37^. Also mice lacking the IκB family member Bcl-3 are more susceptible compared to wild-type mice to *T. gondii*^38^. Our results indicate that the assessment of NF-κB activation might have a prognostic value to help assess the immunogenicity of newly synthesized recombinant proteins as described by others^39^, however this assumption undoubtedly requires further evaluation with many more antigens, to draw any conclusion.

In summary, the immune response induced by vaccination with recombinant chimeric *T. gondii* proteins SGR and/or SMMG administered with a safe adjuvant resulted in long-term immunity, as demonstrated by the assessed protection rates, defined by decrease in cyst burden, even 6 months post-vaccination. These results are especially valuable in regard to the prospective commercial use of developed vaccines when the persistence of a triggered protective immune response is one of the key factors determining the usefulness of the preparation. The duration of immunity after vaccination directly influences the administration schedule and number of booster doses needed to maintain constant protection from infection. The obtained results again underline how important in vivo models are in research on vaccine development, since in vitro, only selected parameters out of the entire complex immune system are tested, while ultimately, the most important and conclusive result is the achieved vaccine protection against parasites in vivo.

## Methods

### Animals

Mice were bred in the animal facility of the Faculty of Biology and Environmental Protection, University of Lodz under specific pathogen-free conditions. Experiments were carried out on male C3H/HeOuJ mice (8–12 weeks of age) from Charles River Laboratories (Wilmington, MA, USA). Animals (2–3/cage) were kept in stable conditions: temperature of 21 °C+ /−0.5 to + / − 5%, 55% humidity, 12/12–h dark/light cycle, 15–20 air exchanges per hour and had free access to water and standardized feed as described previously^40^. Mice were acclimated for 3–5 days before the start of experimental procedures and monitored throughout the experiment.

All experimental procedures were approved by the Polish Local Ethics Commission for Experiments on Animals in Lodz (Agreement 66/ŁB79/2017) and these were in accordance with the Polish legal act on the welfare of animals used for educational and scientific purposes and conform to European Directive 2010/63/EU of the European Parliament and of the Council of 22 September 2010 on the protection of animals used for scientific purposes. The manuscript was prepared according to the ARRIVE guidelines 2.0^41^.

### Parasites

The *T. gondii* DX strain was used to cause chronic parasite infection in experimentally vaccinated and control (PBS) C3H/HeOuJ mice. This strain was maintained in vivo in both C3H/HeOuJ and BALB/c mice. A highly virulent RH strain (50174, ATCC, Manassas, VA, USA) maintained in vivo and a Me49 (50611, ATCC, Manassas, VA, USA) strain maintained in vitro on human foreskin fibroblasts (Hs27, CRL-1634, ATCC, Manassas, VA, USA) were used to obtain water soluble, whole-cell tachyzoite lysate antigens (TLA) with the freeze–thaw technique, as described previously^42,43^.

### Production of recombinant chimeric proteins

Tri- and tetravalent chimeric *T. gondii* recombinant proteins SAG2-GRA1-ROP1 (SGR) and SAG1-MIC1-MAG1-GRA2 (SMMG) were produced as a result of the expression of fusion genes composed of three or four different gene fragments encoding various immunodominant fragments of *T. gondii* antigens (for SGR amino acid residues 31–170 of SAG2, 26–190 of GRA1, and 85–396 of ROP1 and SMMG amino acid residues 49–311 of SAG1, 25–182 of MIC1, 30–202 of MAG1, and 51–185 of GRA2), as presented in Supplementary Fig. 3. Antigens were obtained according to procedures described previously^32,44^. Briefly, the recombinant proteins SGR and SMMG were expressed as insoluble proteins with calculated molecular masses of 72.21 and 83.61 kDa, respectively. The bacterial cells were harvested by centrifugation (5000×*g*, 10 min, 4 °C), and the pellets from 100 ml of culture were resuspended in 30 ml of buffer containing: 5 M urea, 20 mM Tris, 0.5 M NaCl, 5 mM imidazole, 0.1% Triton X-100, pH 7.9 and sonicated. After centrifugation (12,000×*g*, 30 min, 4 °C), the protein extracts, obtained by dissolving the inclusion bodies (using 5 M urea), were purified by Ni^2+^-iminodiacetic acid-Sepharose column in accordance with the manufacturer's instructions (Novagen, Merck KGaA, Darmstadt, Germany). Proteins were eluted from the column using buffer without denaturing agent (urea) containing: 20 mM Tris, 0.5 M NaCl, 0.5 M imidazole, 0.1% Triton X-100, pH 7.9. The expression system produced approximately 31 and 24 mg of purified proteins from a liter of induced *Escherichia coli* Rosetta(DE3)pLacI (Novagen, Merck KGaA, Darmstadt, Germany) culture for SGR and SMMG, respectively. The purification resulted in electrophoretically homogeneous protein preparations with a purity above 95%.

### In vitro monocytes stimulation

Before performing test, recombinant proteins were tested for their possible endotoxin contamination after the purification, which may lead to false positive results, using commercially available Pierce Chromogenic Endotoxin Quant Kit (Thermo Fisher Scientific, Waltham, MA, USA), based on Limulus Amoebocyte Lysate LAL method. To assess the recombinant antigens potential to elicit a proinflammatory response of monocytes and macrophages, that are important as APC cells in vivo, to induce specific T cells response, we used modified THP1-Blue (InvivoGen, San Diego, USA) human monocytes with NF-κB-inducible SEAP reporter construct. Experiments were performed according to manufacturer’s protocol, with modifications. Briefly, cells were maintained in RPMI 1640 medium (Gibco Thermo Fisher Scientific, Waltham, MA, USA), with 2 mM l-glutamine, 25 mM HEPES, 10% heat-inactivated fetal bovine serum (ATCC, Manassas, VA, USA), 100 μg/ml normocin (InvivoGen, San Diego, USA), penicillin–streptomycin (100 U/ml-100 μg/ml) (Merck KGaA, Darmstadt, Germany), 10 μg /ml blasticidin (InvivoGen, San Diego, USA) as a repressor, in 37 °C, 5% CO_2_. For tests medium without blasticidin was used according to the cell manufacturer's protocol. QUANTI-Blue (InvivoGen, San Diego, USA) reagent was used as a chromogen to determine any alkaline phosphatase activity in a biological sample. *E. coli* O55:B5 LPS (Merck KGaA, Darmstadt, Germany) was used as a positive control in concentration of 5 ng/ml. Cells alone (medium) were used as a negative control. For test, 96 well tissue culture plates were coated using 5 μg of recombinant antigens per well in 200 μl of sterile coating buffer (0.1 M Sodium Carbonate, pH 9.5) in 4 °C overnight. Medium and LPS wells were also treated with coating buffer prior to the cells seeding. Experiment was performed in 6 wells per each stimuli, and 12 wells for medium. Wells were washed 3 × with sterile PBS before cells addition. Cells were seeded at density of 9 × 10^4^ per well, according to the manufacturer’s protocol, and stimulated for 24 h. After incubation 20 μl of each cell supernatant was added to 180 μl of QUANTI-Blue reagent on microplate and incubated for 6 h in 37 °C. After incubation optical density at 650 nm was read on microplate reader. The viability of cells after stimulation was also assessed using Alamar Blue assay to avoid false negative results caused by possible protein cytotoxicity.

### Mouse immunization and challenge

**Figure 6:**
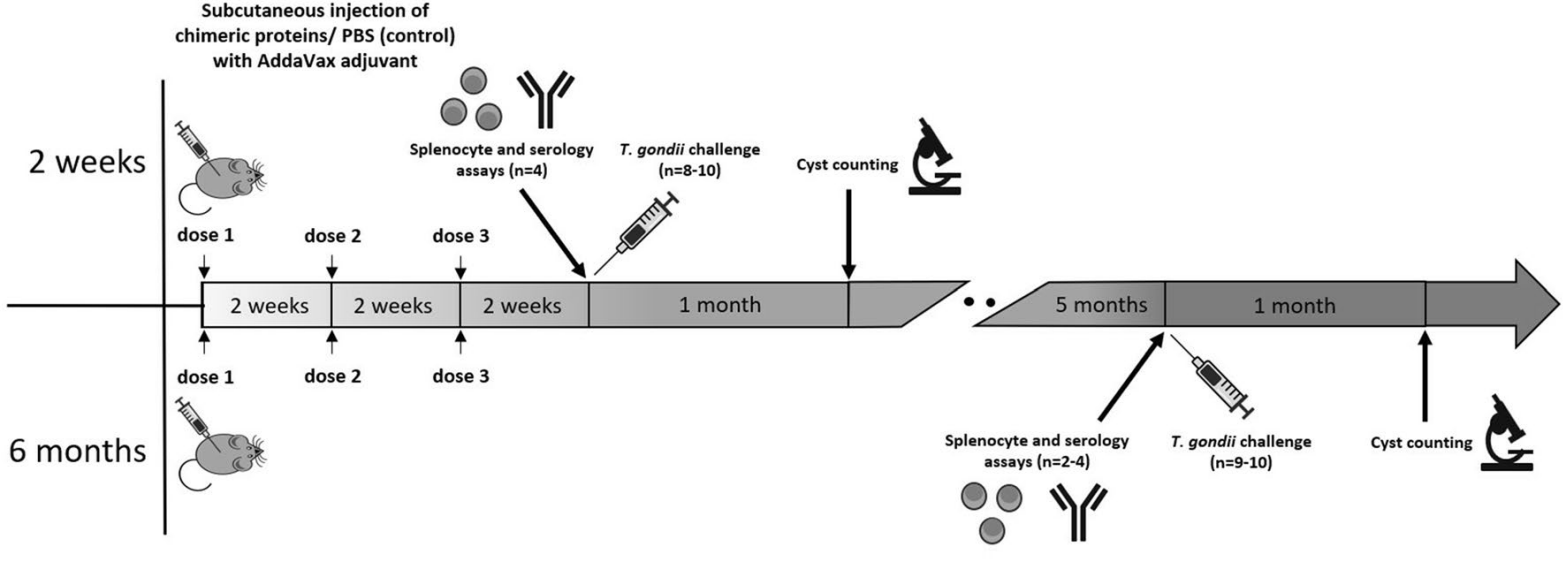
Mouse immunization and challenge protocol.

Mice were first randomly allocated to cages, which were then randomly assigned to four groups (4 mice per group for the splenocyte/serology assay and 8–10 for the *T. gondii* challenge) and subcutaneously vaccinated three times at two-week intervals with SGR (10 µg/mouse), SMMG (10 µg/mouse), SGR + SMMG (10 + 10 µg/mouse), or PBS (control). Chimeric proteins/PBS were emulsified, according to the manufacturer’s protocol, in squalene-based oil-in-water nanoemulsion AddaVax (InvivoGen, San Diego, USA). All mice under study, regardless of the group (control/immunized), underwent the procedures at the same time. All experiments on animals were performed by the same experimenters and only one of them was aware of mice allocation. Two weeks or 6 months after the last dose, mice were anesthetized with sodium pentobarbital (intraperitoneal injection, 200 mg/kg) and euthanized by cervical dislocation for subsequent removal of organs and tissues. Obtained blood/spleens were used for serology/splenocyte assays. Remaining animals were challenged intraperitoneally with 5 *T. gondii* DX cysts. Mouse sera were stored at − 20 °C. Immunoprotection achieved by vaccination was assessed one month after experimental infection. For that purpose the isolated brains were mechanically homogenized in PBS at final volume of 2.5 ml and stored at + 4 °C. Next, in 25 µl samples of each homogenate, in duplicate, the number of *T. gondii* cysts was determined using inverted optical microscope. Based on the mean value the total cyst load for 2.5 ml of the homogenate, corresponding to the whole mouse brain, was calculated. The vaccination and the experiments schedule is shown in the diagram (Fig. 6).

### Serology

Two weeks and 6 months after the last boost immunization, immunoglobulin levels were determined in diluted mouse sera: 1:100 for IgG specific to TLA of the RH and Me49 strains and from 1:100 to 1:204,800 for IgG2a and IgG1 specific to vaccine antigens using an ELISA test (Enzyme-Linked Immunosorbent Assay). Assays were performed as described previously^42^. Briefly, 96-well MaxiSorp plates (Thermo Fisher Scientific, Waltham, MA, USA) were coated overnight with 100 µl of TLA or recombinant antigens at a final concentration of 2 µg/well. The wells were then blocked with PBS supplemented with 10% of FBS (Merck KGaA, Darmstadt, Germany) and next diluted sera were added in duplicate. The reaction was developed with the secondary HRP-conjugated antibodies: goat anti-mouse IgG (Jackson ImmunoResearch, West Grove, PA, USA), goat anti-mouse IgG1 (Bio-Rad, Hercules, CA, USA), goat anti-mouse IgG2a (Bio-Rad, Hercules, CA, USA) and the 2,2-azino-bis(3-ethyl-benzothiazoline6-sulfonic acid) diammonium salt (ABTS) (Merck KGaA, Darmstadt, Germany) serving as a chromogen. The absorbance was measured at 405 nm (Multiskan EX automatic ELISA reader) (Thermo Fisher Scientific, Waltham, MA, USA) and IgG1 and IgG2a titers were defined, as the highest serum dilution with OD > 0.3.

### Splenocyte assay

A splenocyte assay was performed as previously^32,42^. Briefly, spleens from immunized and control mice were isolated, and single-cell suspensions of splenocytes were obtained by mechanical homogenization followed by erythrocyte lysis. The number and viability of splenocytes were determined by the trypan blue exclusion method using TC20 automated cell counter (Bio-Rad, Hercules, CA, USA). Cells were of each mice were cultured in Iscove’s modified Dulbecco’s medium (IMDM) (Cytogen, Zgierz, Poland), supplemented with 5% of heat inactivated FBS, 100 U/ml penicillin + 100 µg/ml of streptomycin (Merck KGaA, Darmstadt, Germany) and stimulated in triplicate for each mice using the previously obtained native *T. gondii* RH strain antigen TLA at a final concentration of 10 µg/ml. Concanavalin A at the final concentration of 2.5 µg/ml (Merck KGaA, Darmstadt, Germany) served as a positive control, while culture medium alone served as a negative control. After 48 h (IL-2) or 72 h (IL-10, IFN-γ) of incubation at 37 °C in a humid atmosphere with 10% of CO_2_, the supernatants were collected and stored at − 80°C, to assess the concentrations of secreted cytokines using commercially available OptEIA ELISA sets (BD Biosciences, San Jose, CA, USA), based on the log–log regression analysis, according to the manufacturer’s instructions. For statistical analysis, cytokine production values from stimulated wells were averaged, thus plotted values represent the average production of the individual mouse.

### Statistical analysis

Graphs and all statistical analyses were performed using GraphPad Prism 9.4.1 for Windows (Dotmatics, GraphPad Software, San Diego, California, USA). The Shapiro–Wilk test was used to assess the Gaussian distribution. The Brown-Forsythe test was used to verify equality of group variances. Aside from the cyst number, the results are presented as the group mean value and its standard deviation SD. Antibody titers were transformed logarithmically to attain normality. Results that met criteria of parametric tests, such as activation of NF-κB pathway in human monocytes and antibody titers, were proceeded using analysis of variance ANOVA test. Post-hoc tests used for each analysis are described under each graph. The nonparametric Kruskal-Walli’s test followed by Two-stage linear step-up procedure of Benjamini, Krieger and Yekutieli post hoc, was used in case of the data that did not fit the parametric methods, such as IgG antibodies level, cytokine assays and cyst burden. In case of all statistical analysis performed alpha α was set to 0.05.

### Supplementary Information

Supplementary Figures.

## Data Availability

All data generated and analyzed during this study that supports the finding are included in this published article/Supplementary Materials. Further inquiries can be directed to the corresponding author.
