# Supplementary material for: Evaluation of long-term immunity and protection against T. gondii after immunization with multivalent recombinant chimeric T. gondii proteins
Source: Sci Rep. 2023 Aug 10;13:12976. doi: 10.1038/s41598-023-40147-z (PMC10415312; doi:10.1038/s41598-023-40147-z)
Supplement: Supplementary file 1 — Supplementary Figures. [file 41598_2023_40147_MOESM1_ESM.docx]

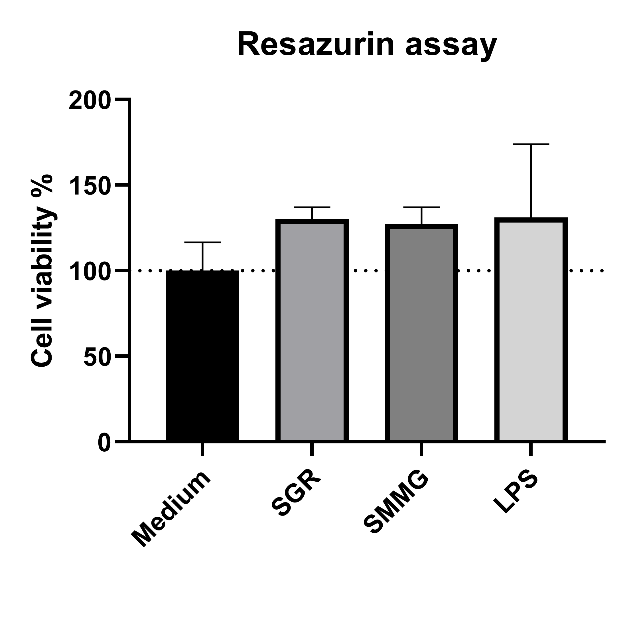


**sFigure 1.** Resazurin assay of THP1-Blue monocytes after stimulation with recombinant proteins. Percent of cell viability was calculated based on 550/600 OD read.

**
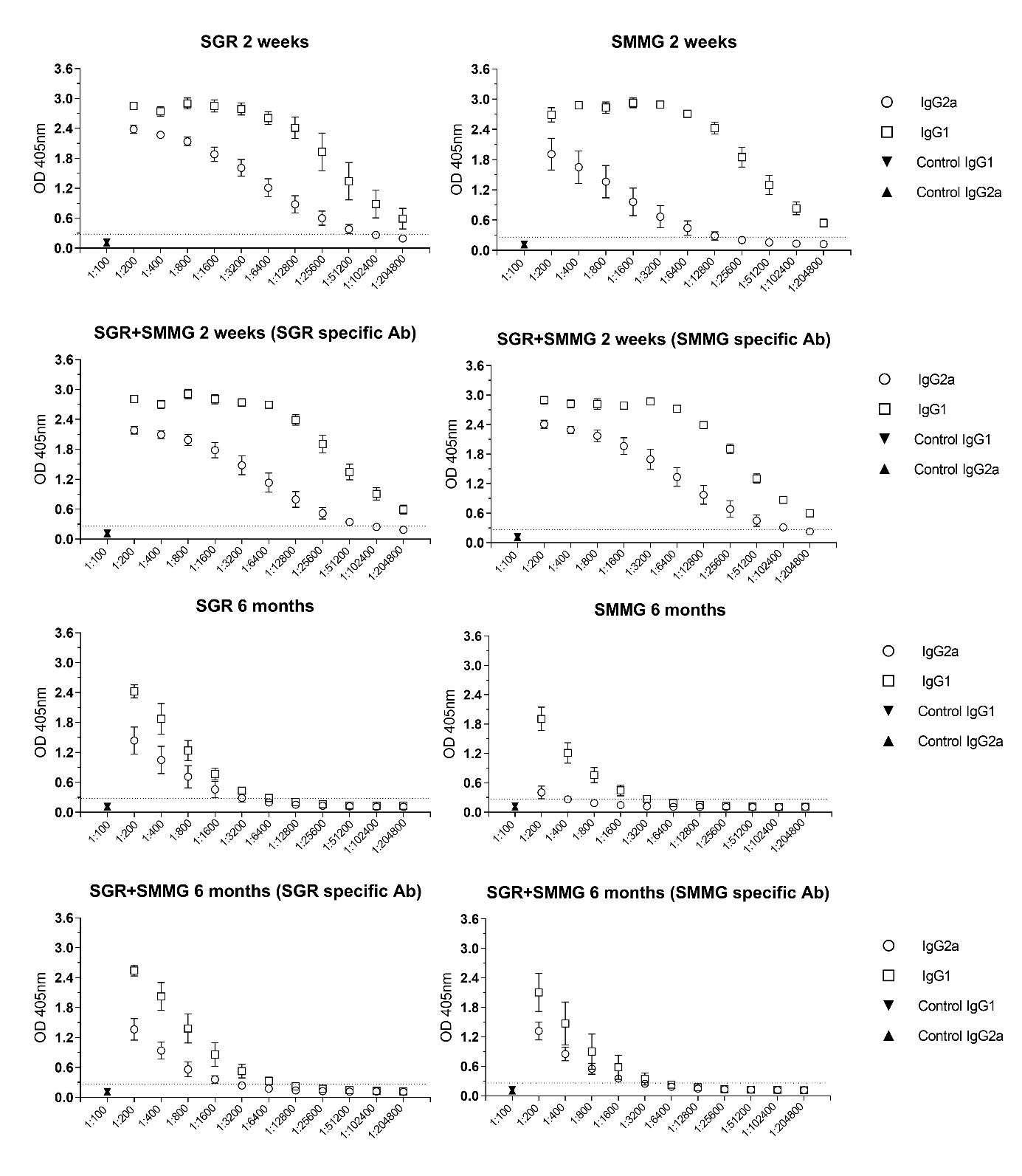
**

**sFigure 2.** Reactivity of IgG1 and IgG2a antibodies specific to antigen used for vaccination in dilutions ranging from 1:100 to 1:204800. 0.3 OD threshold was marked, as an indicator for titer determination.

**
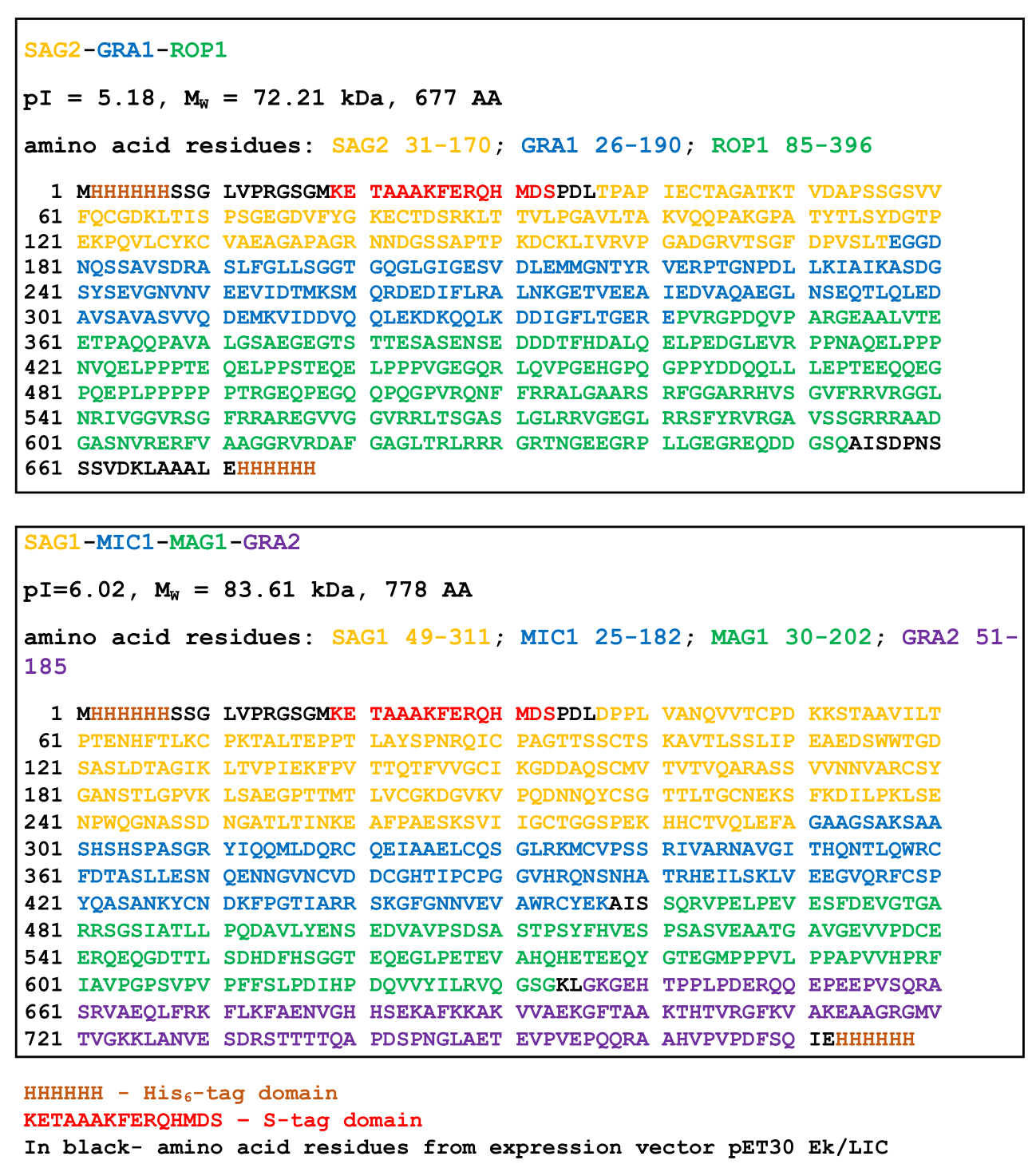
**

**sFigure 3.** Schematic amino acid sequence of tested recombinant chimeric *T. gondii* proteins.
